# Anti-*Chlamydia trachomatis* Host Defence Arsenal Within the Cervicovaginal Environment

**DOI:** 10.3390/ijms27021115

**Published:** 2026-01-22

**Authors:** Simone Filardo, Giulia Chicarella, Rosa Sessa, Marisa Di Pietro

**Affiliations:** 1 Regional Department of Clinical Microbiology, University Hospital Waterford, X91 ER8E Waterford, Ireland; 2 Department of Public Health and Infectious Diseases, “Sapienza” University, 00185 Rome, Italy; giuliachica57@gmail.com (G.C.); rosa.sessa@uniroma1.it (R.S.)

**Keywords:** *Chlamydia trachomatis*, cervicovaginal environment, host defence factors

## Abstract

*Chlamydia trachomatis* has a significant impact on public health, especially among adolescents and young women; it primarily affects urogenital epithelial cells, leading to cervicitis and urethritis, with >90% of cases showing no symptoms. Consequently, chlamydial infections are commonly misdiagnosed, and, if untreated, they may result in severe reproductive sequelae including infertility. A better understanding of *C. trachomatis* cell biology and bacterial–host cell interactions may be helpful to identify strategies able to counter its transmission among the population, as well as its dissemination in reproductive tissues, reducing the risk of developing severe reproductive sequelae. Therefore, the present review aims to summarize the evidence on the interplay between *C. trachomatis* and the host defence factors within the cervicovaginal environment. The sophisticated strategies employed by this clinically significant pathogen to counteract these mechanisms are also discussed. In the literature, the main defence factors include the microbiota dominated by *Lactobacillus crispatus* and several molecules like lactoferrin, able to protect the cervicovaginal microenvironment against *C. trachomatis* through several mechanisms (e.g., EB coaggregation and competitive exclusion, as well as anti-inflammatory activity). However, the major player in clearing chlamydial infections remains the interferon-gamma (IFN-γ) produced by natural killer and T cells, via the depletion of critical nutrients for *C. trachomatis* such as tryptophan, or via the ubiquitylation and destruction of chlamydial inclusions.

## 1. Introduction

*Chlamydia trachomatis*, the leading cause worldwide of sexually transmitted bacterial diseases in humans, has significant public health implications, particularly for women of reproductive age. According to the 2020 World Health Organization’s report, approximately 129 million new cases of *C. trachomatis* occur each year, with young people and adolescents being particularly vulnerable to chlamydial infections; in 2023, the highest rate of *C. trachomatis* cases was reported among young women aged 15–24 years in the United States (15–19 years: 3435.1 per 100,000 population; 20–24 years: 2566.1 per 100,000 population) and in Europe (15–19 years: 400 per 100,000 population; 20–24 years: 700 per 100,000 population) [1,2].

In women, *C. trachomatis* primarily affects urogenital epithelial cells, leading to cervicitis and urethritis, with >90% of cases showing no symptoms. Consequently, chlamydial infections are commonly misdiagnosed and, if untreated, may result in severe reproductive sequelae including pelvic inflammatory disease, ectopic pregnancy and obstructive infertility. Furthermore, *C. trachomatis* genital infections also play a role in the acquisition of other sexually transmitted infections, such as HIV, and seem to be involved in the pathogenesis of HPV-mediated cervical cancer [3,4]. In addition to genital infections, *C. trachomatis* has harmful effects on pregnancy outcomes and neonatal health, including pre-term birth, stillbirth and low-birthweight babies [5,6].

In recent years, several studies have evidenced genes and mutations associated with resistance to antibiotics in all Chlamydia species, opening the possibility that these intracellular obligate pathogens, including *C. trachomatis*, may develop antibiotic resistance. However, to date, *C. trachomatis* continues to exhibit susceptibility to first- and second-line antibiotics, and the only convincing demonstration of gained resistance to an antibiotic commonly used for treating Chlamydia infections has been observed in *Chlamydia suis*; specifically, the acquisition of the *tetC* gene cassette by *C. suis* has been described as a consequence of the large use of tetracyclines as growth promoters in poultry, swine and cattle in Europe [7,8,9].

In this scenario, a greater understanding of *C. trachomatis* cell biology and bacterial–host cell interactions is of particular importance to identify strategies able to counter its transmission among the population, as well as its dissemination in reproductive tissues, reducing the risk for developing severe reproductive sequelae. Therefore, the present review aims to summarize the evidence on the interplay between *C. trachomatis* and the host defence factors within the cervicovaginal environment. The sophisticated strategies employed by this clinically significant pathogen to counteract these mechanisms are also discussed.

## 2. *C. trachomatis* Interaction and Host Defence Factors

### 2.1. C. trachomatis Developmental Cycle

*C. trachomatis*, a Gram-negative obligate intracellular bacterium, has a unique biphasic developmental cycle characterized by two morphologically and functionally distinct forms: the elementary body (EB) and the reticulate body (RB). The EB is the extracellular infectious form, classically considered metabolically inactive, whereas the RB is the intracellular, metabolically active, replicative form.

The developmental cycle begins when EBs attach and enter the host cell by endocytosis. It is thought that the interaction of EBs with the host cell occurs in a two-step process involving a reversible interaction with heparan sulphate glycosaminoglycan (GAG) receptors, followed by an irreversible binding to the cell surface via membrane proteins. Several *C. trachomatis* adhesins, including outer membrane complex protein B (OmcB) and the major outer membrane protein (MOMP), are able to bind directly, or via heparan sulphate, to GAG receptors. Other chlamydial adhesins and host receptors involved in the adhesion of *C. trachomatis* to the host cell are polymorphic membrane proteins (Pmps) and human epidermal growth factor receptors. Also, *C. trachomatis* lipopolysaccharide (LPS) has been described to interact with the host cell cystic fibrosis transmembrane conductance regulator (CFTR), resulting in the uptake of the pathogen within the host cell. Soon after their attachment to the host cell membrane, EBs are internalized and confined to a vacuole termed an inclusion, through a process requiring the secretion of Type III secretion system (T3SS) effector proteins; the translocated actin-recruiting phosphoprotein and translocated membrane-associated effector A have been described to modulate the host cell actin. Then, the chlamydial inclusion is decorated with T3SS proteins, known as inclusion membrane (Inc) proteins, that subvert host cellular processes to establish the infection and promote its survival. Indeed, Incs play a key role in nutrient acquisition through the recruitment of Golgi and endoplasmic reticulum vesicles, as well as in the escape of EB-containing endosomes from the endo-lysosomal pathway. In addition to Incs, other chlamydial proteins, like the chlamydia protease-like activity factor (CPAF) and the high-temperature requirement A protein (HtrA), have an important role in maintaining the integrity of the inclusion and in promoting the intracellular survival of *C. trachomatis* [10,11]. Within the inclusion, EBs shift to RBs, which replicate and, after several rounds of replication, differentiate back into EBs. Interestingly, several experiments have demonstrated that *C. trachomatis* divides by a polarized budding mechanism within the inclusion [12,13,14,15,16]. After approximately 48–72 h, the EBs, released by inclusion extrusion or cell lysis, spread and infect neighbouring epithelial cells, perpetuating the infectious process [10].

Over the years, in vitro studies have suggested that *C. trachomatis* can enter into a persistence state after exposure to stress conditions such as treatment with penicillin, interferon-gamma (IFN-γ), or iron depletion; RBs become enlarged, leading to atypical forms, namely persistent forms, with no production of infectious progeny. Following the removal of the inducer, the persistent forms can transition back into RBs, resuming the developmental cycle. Persistent forms have been described to be more suited to evading the host immune response and harder to eradicate with antibiotics; as a result, they may contribute to the chronic inflammatory state and consequent tissue damage underlying the severe reproductive sequelae related to *C. trachomatis* urogenital infections [17]. Despite extensive in vitro evidence on *C. trachomatis* persistent forms, there is a paucity of in vivo studies demonstrating their detection in the human genital tract [18,19,20].

### 2.2. Cervicovaginal Defence Factors Against C. trachomatis

**Figure 1:**
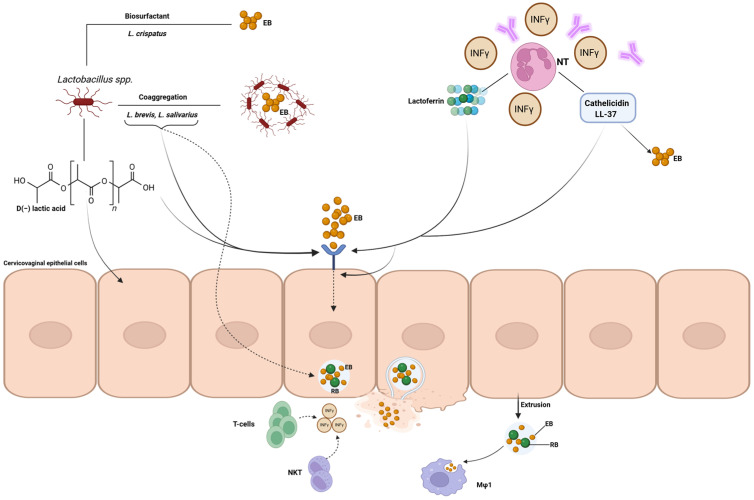
A schematic representation illustrating how the cervicovaginal host defence arsenal can counter *C. trachomatis* infection. *L. crispatus*, *L. salivarius* and *L. brevis* have adverse effects on elementary chlamydial bodies (coaggregation, biosurfactant production), on chlamydial adsorption to epithelial cells (competitive exclusion or reduced exposure and expression of the host receptor) and on the intracellular phases of chlamydial replication; *L. crispatus* also shows anti-chlamydial activity by inhibiting epithelial proliferation and cell-cycle regulation by D(−) lactic acid production. Lactoferrin inhibits the early phases (adhesion and invasion) of *C. trachomatis* infection, whereas cathelicidin LL37 inactivates EBs or prevents their entry into the host cell, as well as their intracellular growth. Neutrophils, in the presence of IFN-γ and anti-Chlamydia antibodies, and IFN-γ/LPS-induced macrophages (M1-type macrophages) can kill *C. trachomatis*. IFN-γ produced by T and natural killer cells can kill *C. trachomatis* via depletion of tryptophan and the ubiquitylation and destruction of chlamydial inclusions. NKT, natural killer cell; Mφ, macrophage.

In the female genital tract, the anti-chlamydial host arsenal involves the cervicovaginal microbiota, various proteins and the immune system (Figure 1).

#### 2.2.1. Cervicovaginal Microbiota

The cervicovaginal microbiota of healthy women is dominated by facultative anaerobes, mostly represented by *Lactobacillus* species. On average, the concentration of lactobacilli is between 10^7^ and 10^9^ colony-forming units (CFUs) per gram of vaginal secretion, while other bacteria, such as *Prevotella* spp., *Peptostreptococcus* spp., *Atopobium vaginae* and *Gardnerella vaginalis*, are present in low abundance [21].

Over the years, a growing body of evidence has shown that the cervicovaginal microbiota composition is highly dynamic and affected by different factors, including age, ethnicity, lifestyle, hormonal fluctuations, poor hygiene practices, antibiotic and/or hormonal therapies, probiotics and the activity of the immune system. Recent evidence has also suggested that increased psychological stress, the consumption of processed food rich in fat and carbohydrates, and high levels of urbanization may be emerging factors impacting the cervicovaginal microbiota composition [22].

Thanks to the development of metagenomic approaches based on the sequencing of the bacterial 16s rRNA gene via Next-Generation Sequencing, it is currently possible to perform more accurate profiling of the human cervicovaginal microbiota composition. Nowadays, five Community State Types (CSTs) based on the dominant lactobacillus species have been described [23]. CST I and CST II are dominated by *Lactobacillus crispatus* and *Lactobacillus gasseri*, respectively; both *Lactobacillus* species are known for their strong protective properties and the maintenance of a low vaginal pH. CST V is characterized by a high abundance of *Lactobacillus jensenii*, also considered a stable and beneficial commensal. On the contrary, CST III, dominated by *Lactobacillus iners*, has been described to play an ambiguous role in the maintenance of cervicovaginal health, since *L. iners* has been found in both healthy cervicovaginal environments and pathological conditions [24]. Lastly, CST IV is characterized by a scarcity of *Lactobacillus* spp. and the presence of a diverse array of strict and facultative anaerobes such as *Gardnerella vaginalis*, *Atopobium vaginae* and *Prevotella* spp. [23,25,26]. Clinically, CST IV is referred to as bacterial vaginosis (BV), which is considered the most common pathological condition globally among reproductive-age women, with an estimated prevalence of 23–29%. In addition, BV has been associated with an increased risk for the acquisition of sexually transmitted infections [27].

**Figure 2:**
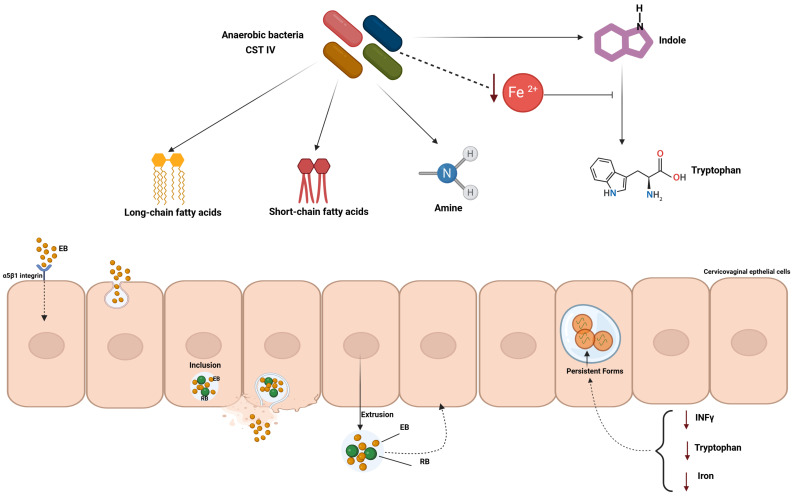
A schematic representation illustrating cervicovaginal risk factors for *C. trachomatis* infection. A microbiota dominated by a diverse mix of anaerobes (CST IV), clinically referred to as bacterial vaginosis, can produce several key metabolites, such as indole and long-chain fatty acids, for *C. trachomatis* intracellular growth and maintaining inclusions. Anaerobic bacteria also metabolize iron, leading to iron depletion and, hence, inducing *C. trachomatis* to switch to the tryptophan salvage pathway by increasing the production of tryptophan synthase. In the presence of low levels of iron, tryptophan, or IFN-γ (during chronic infection), *C. trachomatis* can generate persistent forms, responsible for the inflammatory state underlying tissue damage.

To date, clinical metagenomic studies on cervicovaginal microbiota show that the dominant bacteria in healthy women are *L. crispatus* (CST I), followed by *L. gasseri* (CST II) and *L. jensenii* (CST V). By contrast, a cervicovaginal microbiota dominated by *L. iners* (CST III) or by a diverse mix of anaerobes (CST IV) is associated with *C. trachomatis* infection [28,29]. The high rate of *C. trachomatis* infection in women with a CST IV microbiota can be correlated to the presence of anaerobes like *Prevotella* spp.; these are able to produce indole, an intermediate of tryptophan, which is a well-known key nutrient for the intracellular growth of *Chlamydia*. *C. trachomatis* cannot synthesize tryptophan de novo, and urogenital strains express a functional tryptophan synthase, encoded by the *trpBA* genes, which allows them to convert indole into tryptophan [30]. Concomitantly, the cervicovaginal environment is likely poor in iron, another critical nutrient required by *C. trachomatis* [31]; under these conditions, *C. trachomatis* may switch to the tryptophan salvage pathway by increasing the production of tryptophan synthase, though this interesting hypothesis has not been tested in vivo [32,33] (Figure 2).

Additionally, metabolome studies identified distinct metabolic features (cadaverine, putrescine, and long-chain fatty acids like decanoic, pentadecanoic, heptadecanoic, arachidic, behenic, cerotic and myristic acid) and some metabolites, such as oleic acid, as being important for chlamydial genital infection [34]. Indeed, in the literature, there is evidence that *C. trachomatis* is dependent on long-chain fatty acids, mainly oleic acid, for maintaining the inclusions and sustaining the infection [35]. Metabolome and microbiome analyses alongside in vitro studies evidenced that cervicovaginal fluid containing a CST IV microbiota, biogenic amines (e.g., putrescine and cadaverine) and short-chain acids had poor anti-chlamydial activity [36,37].

The high rate of *C. trachomatis* genital infection related to CST IV microbiota is also supported by in vitro studies highlighting that the biofilm produced by *Gardnerella vaginalis* may be a reservoir of *C. trachomatis*. Specifically, by using an in vitro co-culture transwell-based biofilm model, it has been demonstrated that *C. trachomatis* is able to survive, for up to 72 h, inside the biofilm produced by *G. vaginalis*, retaining its infectious properties [38].

Concerning the protective effect of specific cervicovaginal *Lactobacillus* species against *C. trachomatis*, Gong et al. [39] was the first to demonstrate the anti-chlamydial activity of several standard strains of *L. crispatus*, *L. gasseri* and *L. jensenii* towards *C. trachomatis* EBs through a lactic acid-dependent mechanism. Then, Edwards et al. [40], in 2019, by using a three-dimensional cervical epithelium model, demonstrated the D(−) lactic acid-mediated inhibition of epithelial cell proliferation as a further protective mechanism by which *L. crispatus* and *L. jensenii* were able to reduce *C. trachomatis* infectivity. By contrast, *L. iners*, which produces the L(−) isoform of lactic acid, did not reduce cell proliferation, resulting in lower anti-chlamydial activity [40,41]. Further interesting evidence was the ability of D(−) lactic acid-producing *Lactobacillus* spp. to regulate epigenetic mechanisms, such as histone deacetylase-controlled pathways, leading to a reduction in cell cycling and, hence, reduced *C. trachomatis* vulnerability [40] (Figure 1).

Other protective mechanisms include the ability of several cervicovaginal *Lactobacillus* species to negatively impact the different phases of the *C. trachomatis* developmental cycle. Specifically, *Lactobacillus* spp. have adverse effects on elementary chlamydial bodies, on chlamydial adsorption to epithelial cells and on the intracellular phases of chlamydial replication [42,43,44,45]. For example, *L. crispatus*, *Lactobacillus salivarius* and *Lactobacillus brevis* showed co-aggregation abilities with chlamydial elementary bodies, as well as inhibitory activity towards their adhesion to the host cell surface, thus reducing *C. trachomatis* infectivity [42,43,44,45]. In addition, *L. crispatus* clinical isolates reduced *C. trachomatis* infectivity via the production of a biosurfactant; also, fatty acids (pentadecanoic acid, myristic acid, β-hydroxy-myristic acid, β-hydroxy-palmitic acid) present within the lipopeptidic structure of the biosurfactant showed anti-chlamydial activity [46]. Lastly, *L. crispatus* reduced *C. trachomatis* infectivity in cervical epithelial cells by altering the lipid composition of the host cell membrane, as well as reducing both the exposure and expression of the α5 subunit of α5β1 integrin, a crucial receptor for Chlamydia entry into the host cell [47]. Recent observations of downregulation of the *ITGA5* gene encoding the α5β1 integrin, and of other genes involved in cell-cycle regulation (*CCND1*, *CDKN1A* and *HER-1*), suggest that changes in the state of histone lactylation may represent a further anti-Chlamydia epigenetic pathway [42] (Figure 1).

In addition to a direct anti-chlamydial effect of cervicovaginal *Lactobacillus* spp., there is also evidence that they may indirectly counter chlamydial genital infection by, for example, limiting the availability of metabolites essential for chlamydial growth, such as glucose. In support of this mechanism, low levels of glucose were found in cervicovaginal fluid collected from women with a microbiota enriched in *L. crispatus*, possessing anti-chlamydial activity [36,44].

#### 2.2.2. Cervicovaginal Proteins

Among the several immune factors present within the cervicovaginal environment, there is lactoferrin, an 80 kDa multifunctional cationic glycoprotein belonging to the transferrin family. It possesses iron-binding capacity, and it is predominantly found in milk and, to a lesser extent, in the cervical mucus, where it is released by mucosal epithelial cells and neutrophils following an infection [48]. In *C. trachomatis*-infected women, lactoferrin levels were higher when compared to those in uninfected women [49,50,51]. The protective role of lactoferrin against *C. trachomatis* was mostly evidenced by in vitro studies demonstrating its ability to inhibit *C. trachomatis* entry into host cells when incubated with cervical cell monolayers before or at the moment of the infection [43,52]. As a possible anti-chlamydial mechanism, lactoferrin was hypothesized to bind to cell surface glycosaminoglycans and to heparan sulphate proteoglycans [53,54], recognized as potential receptors for *C. trachomatis* adhesion [55]. Particularly interesting is the interplay of lactoferrin/*L. brevis* in inhibiting the early phases of *C. trachomatis* infection of cervical epithelial cells, as evidenced by stronger anti-chlamydial activity exerted by their combination. Specifically, different effects of *L. brevis* and lactoferrin on *C. trachomatis* were demonstrated; *L. brevis* inhibited the adhesion while lactoferrin inhibited the internalization of chlamydial EBs into the host cell [43]. In addition to lactoferrin, other host defence peptides, including cathelicidin LL-37 and human β-defensins (hBDs), are present within the cervicovaginal environment. Cathelicidin LL-37, released in the cervicovaginal fluid from genital epithelial cells and/or recruited neutrophils, has been demonstrated to inhibit *C. trachomatis* infection by inactivating EBs or by preventing their entry into the host cell, as well as their intracellular growth [56]. Otherwise, in vitro studies have evidenced the ability of *C. trachomatis* to inhibit hBD-2 production and, at the same time, to increase IL-8 secretion in human uterine epithelial cells [57]. In support of reduced production of hBDs, cervicovaginal samples from reproductive-age women with *C. trachomatis* genital infection had lower levels of hBD-1, hBD-2 and hBD-3 than did those from uninfected women [58].

#### 2.2.3. Immune Response

The female reproductive tract is lined by epithelial cells overlaid by a mucus layer representing an important protection barrier against sexually transmitted infections, such as *C. trachomatis*. Upon breach of this barrier, *C. trachomatis* components or metabolites (membrane lipoprotein/lipopeptide, heat shock protein 60, double-stranded DNA, cyclic di-AMP, etc.) are recognized by genital epithelial cells and innate immune cells via toll-like receptors (TLRs 2,3,4,6,9), cyclic GMP–AMP synthase (cGAS), nucleotide-binding oligomerization domain-like receptors or NOD-like receptors (NLRs), stimulator of interferon genes (STINGs) and CD14. This results in the activation of TLR-mediated MyD-88/NF-_K_B (adapter myeloid differentiation primary response protein 88/nuclear factor-kB) signalling and STING- or NOD-mediated NF-κB signalling, leading to the production of cytokines and chemokines that play a central role in the host defence against *C. trachomatis* [59,60]. Infected epithelial cells release IL-8 that recruits innate immune cells including neutrophils and monocytes at the infection site. Neutrophils and monocytes/macrophages engage free chlamydial EBs or extrusions, inclusion-like structures liberated from non-lytic epithelial cells; however, only neutrophils in the presence of IFN-γ and anti-*Chlamydia* antibodies, and IFN-γ-stimulated macrophages (M1-type macrophages), are able to kill *C. trachomatis* [61,62]. Indeed, neutrophils are professional phagocytic cells that recognize potential pathogenic microbes opsonized by specific IgG or IgA through their interaction with the surface Fc receptors. Regarding *C. trachomatis*, *Chlamydia*-specific antibodies are able to increase neutrophils’ phagocytic killing only in the presence of IFN-γ, rather than alone, although the exact mechanism is unknown [61]. In addition, neutrophil degranulation products such as lactoferrin and cathelicidin, as well as neutrophil extracellular traps—extracellular DNA associated with antimicrobial proteins—have been described to be effective against *C. trachomatis* [61].

Genital tissue is also composed of dendritic cells and T lymphocytes; recent studies using mouse and in vitro models of infections demonstrated that an efficient CD8+ T cell response was not generated upon chlamydial infection of dendritic cells due to the ability of this pathogen to induce cell death via apoptosis [63]. On a different note, IFN-γ-producing CD4+ T cells were strongly associated with a protective response against *C. trachomatis*. However, CD4+ T cells cannot produce IFN-γ during the early stages of infection, and, hence, natural killer cells of the mucosal immune system in the female genital tract have been described to play a critical role in limiting *Chlamydia* infection via early production of IFN-γ [64].

To date, IFN-γ is well known to activate the catabolic depletion of L-tryptophan via indoleamine-2,3-dioxygenase, restricting *C. trachomatis* growth within host cells. More recent research has also shown the ability of IFN-γ to inhibit chlamydial intracellular growth through the depletion of other essential nutrients (glucose; amino acids: glutamate, aspartate, glycine, alanine; Krebs cycle intermediates: citrate, aconitate, α-ketoglutarate; pyrimidine/purine nucleosides: nucleotide triphosphates adenosine triphosphate, cytidine triphosphate, uridine triphosphate), dependent on metabolic transcription factors such as c-Myc and HIF-α. Interestingly, the depletion of c-Myc is able to restrict intracellular chlamydial growth even in the absence of IFN-γ; by contrast, the overexpression of c-Myc protects *C. trachomatis* from IFN-γ-mediated persistence [65,66]. Additionally, IFN-γ has been described to counter *C. trachomatis* infection by inducing pyroptosis in macrophages, a pro-inflammatory type of cell death via nucleotide-binding oligomerization domain-like receptor family pyrin domain-containing 3 (NLRP3) inflammasome [65].

Of pathological and clinical importance is the different immune response observed in mouse models during acute and chronic *C. trachomatis* infections. During acute infection, macrophages are driven to the M1 phenotype with increased production of IFN-γ from both CD4+ T helper type 1 (Th1) cells and CD8+ T cells and consequent control of chlamydial replication and its eradication. Otherwise, during a chronic infection, the presence of macrophages with an M2 phenotype and reduced numbers of CD4+ and CD8+ T cells secreting mostly immunosuppressive cytokines (Transforming growth factor β and Interleukin-10), instead of IFN-γ, are observed. As a result, *C. trachomatis* generates persistent forms, and M2 macrophages with low CD40 expression promote the differentiation of CD4+ T helper type 2 (Th2) cells and regulatory T cells, leading to sustained *C. trachomatis* genital infection in mouse models [67].

As chlamydial reinfections are common, immunological memory is vital to generate protection against *C. trachomatis*. Among the memory CD4+ T cells, tissue-resident CD4+ T cells (Trms) have been described as necessary in clearing secondary infection since they can remain in the genital tract after clearing of the infection. As a result, Trms may act as a first line of defence upon re-exposure to *C. trachomatis* since they are able to respond more rapidly to a pathogen than other memory T cell subsets that need to traffic to the tissue to respond. However, mouse models have evidenced that the clearance of secondary *C. trachomatis* infection is dependent on the establishment of Trms, as well as on the recruitment of circulating memory T cells to the upper genital tract mucosa [59].

Of note, *C. trachomatis* has evolved different strategies to evade the host immune response by interfering with host multiple signalling pathways involved in immune recognition, inflammation, and host cell apoptosis and autophagy [62,68]. For example, the translocated early phosphoprotein (TepP), an effector of *C. trachomatis* secreted in the host cell cytoplasm, has been shown to inhibit innate immune sensors such as the TLR2, NOD1 and STING pathways by blocking the production of protective inflammatory cytokines and the subsequent systemic immune response [69]. *C. trachomatis* also secretes CPAF, which is able to cleave the neutrophil surface receptor formyl peptide receptor 2, inhibiting the activation of neutrophils and, hence, the production of reactive oxygen species (ROS), neutrophil extracellular traps and degranulation [61]. Again, *C. trachomatis* can interfere with NF-κB signalling by blocking the release of the NF-kB transcriptional complex, as well as its translocation to the cell nucleus, thus inhibiting the host protective inflammatory response [68]. *C. trachomatis* has also been demonstrated to impair the transport of major histocompatibility complex class I molecules and reduce the antigen cross-presentation of dendritic cells, evidencing the reduced capability of the immune system to generate an effective CD8+ T cell response upon chlamydial infection [70]. Recently, the ability of RBs to pass into uninfected neighbouring cells through tunnelling nanotubes avoiding the extracellular microenvironment and, thus, the immune response has been suggested as a further potential evasion mechanism of *C. trachomatis* [71].

Unlike LPS from other Gram-negative bacteria, chlamydial LPS with its unique structure protects *C. trachomatis* from both the innate, intracellular immune response and the adaptive cytotoxic T cell response by inhibiting apoptosis and pyroptosis in genital epithelial cells, as well as by reducing the activation of dendritic cells and, hence, the priming of the CD8 T cell response. In addition to inhibiting priming, it has been shown that chlamydial LPS may alter indirect MHC class I antigen presentation [72,73].

## 3. Discussion

Important advances in the understanding of *C. trachomatis* interaction with host defence factors have been achieved in the field of genital infections. It has been acknowledged for over a decade that a cervicovaginal microbiota dominated by *Lactobacillus* spp. plays a key role in maintaining homeostasis and protecting women’s reproductive health against sexually transmitted pathogens, including *C. trachomatis*. However, the recent development of metagenomic approaches based on the sequencing of the bacterial 16s rRNA gene has evidenced that not all *Lactobacillus* species have protective activity against *C. trachomatis*. Indeed, a growing number of sequencing studies and in vitro experiments based on 2D models have shown that a cervicovaginal microbiota populated by *L. crispatus* plays a protective role against *C. trachomatis*. By contrast, a cervicovaginal microbiota dominated by *L. iners* (CST III) or by a diverse mix of anaerobes (CST IV) is associated with *C. trachomatis* infection [28,29]. Thanks to metabolomic analyses, several interesting metabolites have been associated with protective or harmful bacterial signatures regarding *C. trachomatis* infection. For example, D (−) lactic acid is associated with *L. crispatus* but not *L. iners*; biogenic amines and short-chain and long-chain fatty acids are related to CST IV microbiota [34,36,37].

In the literature, there is proof of several anti-chlamydial mechanisms attributed to *L. crispatus* or lactoferrin, released by neutrophils, including EB coaggregation and competitive exclusion [39,42,43,44,45,46,47]. The strong anti-chlamydial activity of *L. brevis* in combination with lactoferrin, and the anti-chlamydial epigenetic pathways such as the inhibition of epithelial proliferation and cell-cycle regulation mediated by D (−) lactic acid-producing lactobacilli, evidence the crosstalk between the cervicovaginal microbiota and host defence factors in the fight against *C. trachomatis* infection [40,42,43]. Also, the anti-inflammatory activity related to cervicovaginal-specific *Lactobacillus* species and lactoferrin underscores the critical role of cervicovaginal host defence factors in the pathogenesis of *C. trachomatis* genital infection [74]. However, the major player in clearing infections remains the IFN-γ produced by natural killer cells and T-cells, via the depletion of critical nutrients for *C. trachomatis* such as tryptophan, or via the ubiquitylation-mediated killing of chlamydial inclusions [65].

In spite of the different host defence mechanisms, *C. trachomatis* has evolved successful counter strategies to ensure its long-term relationship with the host. Firstly, *C. trachomatis* urogenital strains are able to bypass tryptophan starvation related to IFN-γ exposure by synthesizing it from indole or from its precursor, chorismate, produced by anaerobes populating the CST IV microbiota [75,76]. Secondly, *C. trachomatis* can generate persistent forms responsible for the chronic inflammatory state underlying tissue damage associated with *C. trachomatis* genital infection [65,66]. Interestingly, in vitro studies have recently described how *C. trachomatis* persistent forms may evade both extracellular and intracellular host defences. Specifically, persistent forms may resist the host cell apoptosis and endosomal maturation pathways by retaining early Inc proteins in their inclusion membrane. In addition, they may inhibit peptidoglycan (PG) synthesis and subsequently fail to release PG-derived muropeptides into the host cell, resulting in a decrease in NOD1/NF-κB-mediated IL-8 production and recruitment of innate immune cells [77]. Thirdly, *C. trachomatis* can escape cervicovaginal immune surveillance via several chlamydial effector molecules; CPAF and HtrA degrade cathelicidin LL-37, an anti-chlamydial peptide released by epithelial cells and neutrophils in response to Chlamydia infection, whereas membrane protein gamma resistance determinant protects chlamydial inclusions from ubiquitination and proteasomal degradation [61,78]. Lastly, the formation of extrusions utilized by *C. trachomatis* to exit epithelial and dendritic cells, as well as macrophages, represents an evasion strategy and a dissemination mechanism to more distant sites, thus promoting the ascension of a genital infection to the reproductive tract [79,80,81]. It is reasonable to hypothesize that all these counterstrategies might be employed by *C. trachomatis* in relation to dynamically changing environmental conditions to favour its survival in the cervicovaginal tissues; as a consequence, *C. trachomatis* may be able to alternate between persistent and re-activated states, increasing the risk of incomplete clearance of chlamydial infection. This may result in a chronic infection responsible for the inflammatory state underlying host tissue damage and fibrosis, ultimately leading to pelvic inflammatory disease and tubal factor infertility. Such a condition may be further worsened by an exacerbated immune response and the presence of a CST IV microbiota. The latter has been described to induce the production of multiple proinflammatory molecules, including interleukin (IL)-6, IL-8, TNF-α, IL-1α, matrix metalloproteinase (MMP)-9, MMP-10 and MMP-1, involved in epithelial barrier disruption by inducing oxidative stress, altering miRNA, and promoting cell-cycle arrest, apoptosis and necrosis [74]. Among the immune cells involved in the immune response towards *C. trachomatis*, neutrophils have been described to have a role in *Chlamydia*-induced pathology [61,82]. By using mouse models, it has been evidenced that increased neutrophil survival, recruitment at the infection site and activation during chlamydial genital infection are associated with an increased risk of *C. muridarum*-associated pathology [61]. Interestingly, in a study including women at increased risk of pelvic inflammatory disease, Wiesenfeld et al. [83] evidenced a correlation between increased levels of neutrophil alpha defensins, a marker of neutrophil activation, in the vaginal tract and the development of endometritis after *C. trachomatis* infection. In vivo studies have shown that neutrophils may also contribute to *C. muridarum*-associated pathology through the secretion of ROS that can directly damage host tissues, promote inflammation by increasing neutrophil activation and activate neutrophil degranulation products, such as MMP-9, involved in scarring and fibrosis of the murine oviduct [61].

In the literature, numerous studies have focused their attention on identifying biomarkers predictive of infection risk and adverse outcomes. Serum anti-*Chlamydia* IgG and cervical anti-*Chlamydia* IgA and IgG have been shown to correlate with reduced cervical burden. In addition, high antibody titres (IgG) reflecting repeated or prolonged exposure were linked with enhanced risk for incident infection [84]. A systematic review and meta-analysis of observational studies revealed low- or moderate-quality evidence for an association of *C. trachomatis* serology (anti-chlamydial MOMP and Hsp60 antibodies) with fertility- and pregnancy-related adverse outcomes [85]. Liu et al. [86] identified IgG to some specific proteins of *C. trachomatis*, such as Pgp3 and CT443, as antibodies with the greatest impact on chlamydial ascension and incident infection. In addition, IgG to Pgp3 was also found to be associated with infertility in U.S. women [87]. Recently, by using machine learning-based analytical pipelines applied to clinical and serum IgG immunoproteomic data, it has been evidenced that serum chlamydial-antigen-specific antibodies and risk factors are insufficient to serve as biomarkers for ascension or incident infection [88].

Nevertheless, the currently available evidence is still too weak to justify the usage of chlamydial serology to predict the risk of any adverse fertility-related or pregnancy outcomes.

## 4. Conclusions

A growing body of evidence on the intricate interactions between cervicovaginal defence factors and *C. trachomatis* highlights host microbiota dominated by *L. crispatus* and immune response as important players affecting the risk of acquiring chlamydial genital infection and developing severe reproductive sequelae in women. In vitro studies provide interesting protective mechanisms related to *L. crispatus* or to its metabolites, as well as to other specific Lactobacillus species, although they are of limited translational relevance due to the simplicity of the models. Indeed, in vitro experiments have relied on using 2D human immortalized cervical epithelial cells that cannot accurately simulate the architecture and physiology of the healthy genital tract characterized by the different types of cells, surface mucus, hormones and microbiota that are integral parts of the female reproductive system and interact with each other. In addition, the cells in 2D models lack the ability to nourish dynamic bacterial communities and are exposed to unphysiological stiffness, as they cannot maintain appropriate cell–cell interactions. In the future, advancing the complexity of in vitro systems that better mimic the cervicovaginal microenvironment will help characterize the complex interaction network between *C. trachomatis* and host defences, thus improving the translational potential of *Chlamydia* research. Going forward, 3D models alongside multi-omics approaches will be an essential step towards the discovery of novel therapeutic targets for this clinically significant pathogen.

## Data Availability

No new data were created or analysed in this study. Data sharing is not applicable to this article.
